# Molecular Phylogeography and Intraspecific Divergences in Siberian Wildrye (*Elymus sibiricus* L.) Wild Populations in China, Inferred From Chloroplast DNA Sequence and cpSSR Markers

**DOI:** 10.3389/fpls.2022.862759

**Published:** 2022-05-19

**Authors:** Yi Xiong, Yanli Xiong, Xin Shu, Qingqing Yu, Xiong Lei, Daxu Li, Jiajun Yan, Shiqie Bai, Xiao Ma

**Affiliations:** ^1^College of Grassland Science and Technology, Sichuan Agricultural University, Chengdu, China; ^2^Sichuan Academy of Grassland Science, Chengdu, China

**Keywords:** *Elymus sibiricus*, phylogenetics, ecological niche, genetic diversity, Qinghai-Tibet Plateau

## Abstract

A detailed understanding of the distribution and degree of genetic variation within a species is important for determining their evolutionary potential, which in return facilitates the development of efficient conservation strategies aimed at preserving adaptive genetic variation. As an important perennial, cool-season grass in temperate Eurasia, increasing attention has been paid to Siberian wildrye (*Elymus sibiricus*) due to its excellent ecological utilization value and forage production potential in China, particularly in the Qinghai–Tibet Plateau (QTP) regions. In this study, we applied two chloroplast (cp) genes (*matK* and *rbcL*), three cp spacer regions (*trnY*-*GUA*∼*trnD*-*GUC*, *atpH*∼*atpF*, and *rps4*∼*trnT*-*UGU*), and six cpSSR markers to the genetic and phylogenetic analysis of 137 wild *E. sibiricus* accessions from 23 natural populations that represent the main distribution regions in China. The results show the highest genetic diversity (*h* = 0.913) and haplotype richness (10 haplotypes) for the QTP population, which indicates QTP as the probable diversity center and geographic origin of *E. sibiricus* in China. Population divergence was high, indicating a significant phylogeographic structure together with a significantly higher N_st_ value (N_st_ > G_st_, *P* < 0.05) at the species level, QTP+XJ (combined populations from QTP and Xinjiang), QTP+NC (combined populations from QTP and North China), and XJ+NC (combined populations from Xinjiang and North China) group levels, respectively. An expansion was revealed in the distributional range of *E. sibiricus* in China from paleo times up to the recent past, while a dramatic range of contraction was predicted for the near future. The predicted main limiting factor for the further spread of *E. sibiricus* is an increasing global mean temperature. We recommend that the combination of Es-cpDNA1 and Es-cpDNA3+4+5 can be used as effective markers for phylogenetic analysis and phylogeographical history analysis of *E. sibiricus*. These findings shed new light on the historical population dynamics of cold-season herbs in the QTP region and the north of China and are of great significance for the future establishment of protection and collection strategies for wild *E. sibiricus* germplasm.

## Introduction

Geographical events and global climate oscillations have led to repeated and drastic changes in the environment, something that has had a great impact on the distribution and genetic structure of plants in different ecological distribution areas (Hewitt, 2000). Phylogeography focuses on the evolution of species and different populations within species, as well as the relationship between modern distribution patterns and climate, geological, and historical events (Avise, 2000). After decades of development, phylogeographic studies have been carried out in almost every biogeographic region of the planet (Garrick et al., 2015). Ecological niche models (ENMs) can provide insights into the geographical distribution of a species and can predict distributional changes by using species occurrence data and the suites of environmental conditions experienced at each occurrence point (Araújo and Peterson, 2012). The ENM is then used to predict the potential distribution areas of different periods based on the environmental data of different historical/future periods (climatic data) (Araújo and Peterson, 2012). Combined with molecular phylogeography, ENMs can evaluate the impact of climate change on a species distribution pattern and can help to reveal the process that has shaped the current distribution patterns of a species (Mellick et al., 2012).

Considering genetic processes, mutation, and the recombination rate, variations maintained within the molecular markers retain the genetic imprint of lineage evolution to varying degrees, and thus can be used as an effective and reliable method to detect the population dynamics of a species during past historical periods (ancient or recent) (Arbogast, 2015). Such genetic information on genetic structures, combined with geological movement, climate change, and fossil data, can be effective in helping find the refugia of organisms during ice ages and in inferring expansion and migration routes after the ice age (Petit et al., 2008). Given the features of uniparental inheritance, chloroplast genetic markers (chloroplast DNA sequences and chloroplast SSR) are more sensitive to population genetic effects, such as genetic drift, and are thus widely recognized in population genetics, biogeographic studies, and phylogeography (Petit et al., 2008). Though *matK* and *rbcL* genes have contributed significantly to the study of phylogeography (Garrick et al., 2015), more than two-thirds of the published studies about phylogeography have been carried out using non-coding regions of the chloroplast genome or combined with nuclear simple sequence repeats (nSSRs) (Garrick et al., 2015). However, the most frequently used non-coding regions, such as *psbA*-*trnH*, *trnL*-*trnF*, and *trnL* introns, possess lower variability (Morris and Shaw, 2018). Another polymorphic marker of particular interest is the chloroplast SSR, hereafter referred to as “cpSSRs,” which are usually located in the non-coding regions of the chloroplast genome and show higher intraspecific variations when present in repeated numbers (Wang et al., 2017). The combination of haplotype variation in the population analyzed by chloroplast DNA and phylogenetic analysis carried out by cpSSR helps to examine whether the structure of phylogeography exists.

The Qinghai–Tibet Plateau (QTP) once acted as a refuge for plants in the north temperate region during the Quaternary glacial period and has been recognized as one of the important places of origin and radiation of modern north temperate plants after the glacial period (Liu J. et al., 2017). As a model species of the *Elymus* genus (Triticeae and Poaceae) and an ancient wild grass species, *Elymus sibiricus* is widely distributed in the Eurasian continent. Its distribution areas in China include Northwest and North China, particularly in the QTP regions (Xiong et al., 2021). The germplasms of *E. sibiricus* located in high-altitude regions may exhibit a more significant response to climate change. Under the influence of global climatic shock during the Quaternary ice age, there may have been clear changes in the population size and distribution range of *E. sibiricus*. The variable climatic factors in different distribution areas of *E. sibiricus* make it worth exploring whether phylogeography differentiation is present in different regions. We have obtained some hotspot regions, including two chloroplast genes (*matK* and *rbcL*) and three cp spacer regions (*trnY-GUA*∼*trnD-GUC*, *atpH*∼*atpF*, and *rps4*∼*trnT-UGU*), by blasting the chloroplast genomes of *E. sibiricus* and its related species *Elymus nutans*. These hotspot regions, combined with six polymorphic cpSSRs, were applied in the present study to explore the genetic diversity and phylogeographic structure of 23 natural populations composed of 137 wild accessions. Further, the ENM prediction based on the records of the occurrence of *E. sibiricus* germplasms was used to detect the environmental factors involved in shaping current and future distribution patterns.

## Results

### Genetic Diversity of *Elymus sibiricus* in China

#### Genetic Diversity Estimated by cpSSR

The polymorphism information content (PIC) values of six cpSSR markers varied from 0.8945 (Es-cpSSR6) to 0.9555 (Es-cpSSR2) with an average of 0.9288 (Table 1). The detected alleles of six primer pairs ranged from 18 (Es-cpSSR6) to 37 (Es-cpSSR2), with an average of 28.5 alleles per primer. High genetic diversity ($\bar{\text{h}}$ = 0.933) and Shannon’s information ($\bar{\text{I}}$ = 2.9629) index values were detected based on these cpSSR markers. All the above-mentioned results demonstrated the high polymorphism and good potential for the utilization of these cpSSRs for the genetic diversity studies of *E. sibiricus*. We detected a high genetic diversity of *E. sibiricus* at the species level, in which the QTP group had the highest diversity, which was reflected by the highest Na, Ne, I, and h values (Table 2).

**TABLE 1:** Polymorphism assessment of six cpSSR primers.

Marker	PIC	*h*	N_a_	N_e_	*I*
Es-cpSSR1	0.9054	0.912	27	11.3408	2.7356
Es-cpSSR2	0.9555	0.957	37	23.3736	3.3393
Es-cpSSR3	0.9436	0.946	33	18.6016	3.1415
Es-cpSSR4	0.9404	0.943	29	17.6235	3.0622
Es-cpSSR5	0.9335	0.937	27	15.8656	2.9641
Es-cpSSR6	0.8945	0.902	18	10.2283	2.5346
Mean	0.9288	0.933	28.5	16.1722	2.9629

*PIC, polymorphic information content; h, Nei’s genetic diversity; N_a_, total number of different alleles per locus; N_e_, number of effective alleles; I, Shannon’s information index.*

**TABLE 2:** Genetic diversity of *Elymus sibiricus* estimated by cpSSR.

Group	*N*	N_a_	N_e_	*I*	*h*
Species	137	119	47.033	2.963	0.933
QTP	72	19.667	12.602	2.665	0.913
NC	36	15.667	10.815	2.451	0.879
XJ	29	14.333	10.293	2.427	0.886

*N, sample size; N_a_, number of alleles per locus; N_e_, number of effective alleles per locus; I, Shannon’s information index; h, Nei’s genetic diversity.*

#### Genetic Diversity Estimated by cpDNA

There were 14, 2, and 35 haplotypes detected from cpDNA regions *matK*, *rbcL*, and “Es-cpDNA3+4+5,” respectively (Table 3). The QTP group had the highest haplotype number and Hd value based on all these cpDNA markers. In addition, the QTP group was also characterized by the highest nucleotide diversity (π).

**TABLE 3:** Genetic diversity of *Elymus sibiricus* estimated by cpDNA.

Group	*N*	*matK*				*rbcL*				Es-cpDNA3+4+5			
				
		*S*	*H*	Hd (std)	1,000 × π (Std)	*S*	*H*	Hd (std)	1000 × π (std)	*S*	*H*	Hd (std)	1,000 × π (std)
QTP	72	5	10	0.834 (0.025)	1.08 (0.34)	3	2	0.263 (0.059)	0.55 (0.12)	9	26	0.920 (0.018)	2.58 (0.14)
XJ	29	3	5	0.527 (0.088)	0.42 (0.31)	0	1	0 (0)	0 (0)	6	11	0.885 (0.043)	2.08 (0.19)
NC	36	6	7	0.543 (0.093)	0.53 (0.46)	3	2	0.157 (0.077)	0.33 (0.16)	6	7	0.716 (0.064)	1.25 (0.21)
Species	137	6	14	0.791 (0.025)	0.88 (0.33)	3	2	0.185 (0.041)	0.39 (0.09)	10	35	0.923 (0.013)	2.54 (0.12)

*N, sample size; S, number of segregating sites (excluding indels); H, number of haplotypes; Hd, haplotype diversity; Std, standard deviation; π, nucleotide diversity.*

### Clustering Analysis and Genetic Structure

#### Cluster Analysis for cpSSRs

**FIGURE 1:**
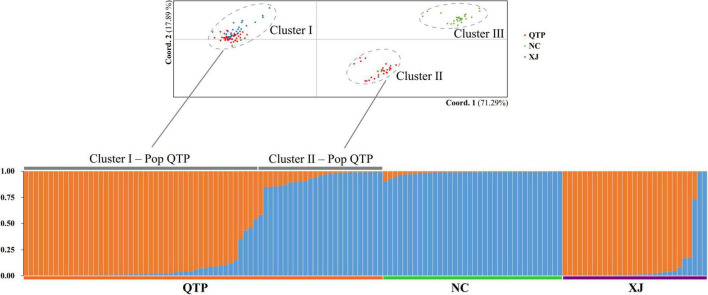
cpSSR genetic structure and principal component cluster results.

Based on the principal component analysis (PCA), we divided all the individuals into three obvious clusters (Figure 1). Cluster III consisted of all NC individuals, while Cluster I and Cluster II were heterogeneous. QTP individuals were divided into two groups, namely, Cluster I-Pop QTP and Cluster II-Pop QTP, in which Cluster II-Pop QTP included all individuals from Tibet (XZ). Bayesian clustering analysis was further applied to clarify the genetic components of *E. sibiricus*. The results suggest that the optimal K value (the number of genetic clusters) for *E. sibiricus* was 2, based on ΔK estimation (Supplementary Figure 1). Individuals in Cluster III had an identical and pure genetic background, which were all from the NC group. However, some individuals from QTP were detected with two genetic backgrounds, in which Cluster I-Pop QTP had a similar genetic constitution to the XJ group, while Cluster II-Pop QTP was more similar to the NC group. Accordingly, the geographical origin of *E. sibiricus* in China could be attributed to the “NC” and “QTP + XJ” groups.

#### cpDNA Haplotype and Phylogenetics

**FIGURE 2:**
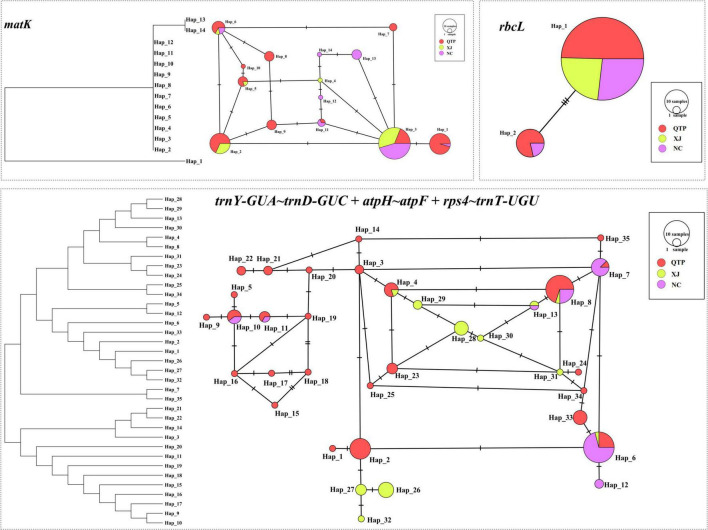
Haplotype network and the phylogenetic tree constructed based on cpDNA datasets.

Three cpDNA regions were applied to connect a haplotype network based on their nucleotide substitutions. The phylogenetic tree was also constructed to reveal the inter-haplotype relationships. As shown in Figure 2, QTP individuals possessed the largest haplotype variation. In addition, the dendrogram indicated the most ancient divergence time of QTP haplotypes.

### Genetic Differentiation and Historical Gene Flow

**FIGURE 3:**
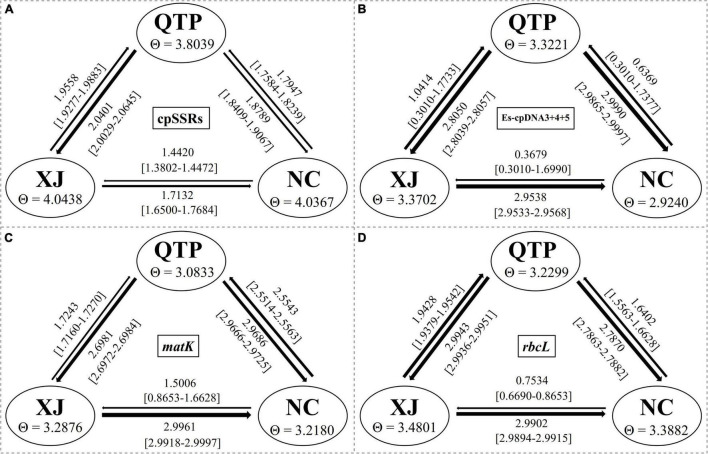
Bidirectional historical gene flow among three groups calculated by Migrate-n. **(A–D)** The results generated by cpSSRs, Es-cpDNA3+4+5, matK, and rbcL respectively.

Analysis of molecular variance (AMOVA) indicated a significant genetic differentiation among the populations based on cpSSRs and cpDNA (Table 4). The pairwise F_*st*_ value further revealed the largest divergence between the NC and XJ groups based on non-functional cp regions (cpSSRs and intergenic cpDNA regions) and the maximum differentiation between QTP and NC or XJ based on functional cp genes (Table 5). G_*st*_ and N_*st*_ values were calculated based on seven levels, namely, species, QTP, XJ, NC, QTP+XJ, QTP+NC, and XJ+NC. The permutation results showed that N_*st*_ was significantly higher than G_*st*_ (*P* < 0.05) when assuming species, QTP+XJ, QTP+NC, and XJ+NC, which indicates a clear phylogeographical structure between pairwise populations (Table 6). Bidirectional historical gene flow was estimated among three groups. It revealed an extensive cp genomic exchange. It is particularly worth noting the obviously higher monodirectional gene flow from the QTP to the non-QTP group and from XJ to NC (Figure 3).

**TABLE 4:** AMOVA analysis inferred by cpDNA and cpSSRs.

Source of variation	df	cpSSRs			*matK*			*rbcL*			Es-cpDNA3+4+5		
					
		SS	%	Fst	SS	%	Fst	SS	%	Fst	SS	%	Fst
Among groups	2	119706.431	64	0.640*	30.240	24	0.241*	3.875	14	0.138*	22.894	17	0.171*
Within groups	134	106371.335	36		142.037	76		33.833	86		160.783	83	
Total	136	226077.766	100		172.277	100		37.708	100		183.677	100	

*Indicates P < 0.001.

**TABLE 5:** Pairwise matrix of population genetic divergence.

Fst	cpSSRs			*matK*			*rbcL*			Es-cpDNA3+4+5		
				
	QTP	NC	XJ	QTP	NC	XJ	QTP	NC	XJ	QTP	NC	XJ
QTP	0.0000			0.0000			0.0000			0.0000		
NC	0.5716	0.0000		0.1983	0.0000		0.0039	0.0000		0.1354	0.0000	
XJ	0.3492	0.7783	0.0000	0.1217	0.0966	0.0000	0.1409	0.0571	0.0000	0.2743	0.4715	0.0000

**TABLE 6:** Detection of a phylogeographic structure based on N_*st*_ and G_*st*_values.

Group	N_*st*_	G_*st*_	N_*st*_ > G_*st*_
Species	0.152	0.048	*P* < 0.05
QTP	0.004	0.002	N.S.
XJ	0.005	0.007	N.S.
NC	0.008	0.011	N.S.
QTP-XJ	0.138	0.060	*P* < 0.05
QTP-NC	0.131	0.022	*P* < 0.05
XJ-NC	0.039	0.026	*P* < 0.05

### Divergence Pattern Driven by Isolation by Distance and Isolation by Environment

The Mantel test was applied to model the correlation between pairwise genetic differentiation (F_*st*_) and geographical or environmental distance. The longitude, latitude, bio-climates, and geographical distance all predicted a significant relationship with F_*st*_ based on cpDNA and cpSSR (Table 7). A significant correlation was also detected between altitude and F_*st*_ based on cpDNA. A generalized linear mixed modeling (GLMM) approach further confirmed a significant correlation between F_*st*_ and bio-climate distance (*F* = 914.566, *P* < 0.01 for cpSSR; *F* = 303.804, *P* < 0.01 for cpDNA) and geographical distance (*F* = 947.372, *P* < 0.01 for cpSSR; *F* = 312.604, *P* < 0.01 for cpDNA) (Table 7). Among the assumed GLMM models for cpDNA, the altitude model (*F* = 278.665, *P* < 0.01) best explained the genetic difference (DIC = −51.576). However, for cpSSR, there was no obvious difference among these models.

**TABLE 7:** Mantel test and the generalized linear mixed modeling (GLMM) between F_*st*_ and geographical (IBD) and bio-climate (IBE) differences.

Source	cpDNA				cpSSR			
		
	r	*P*	F	AIC	r	*P*	F	AIC
Latitude	0.2747	0.00029	307.873**	−33.589	0.125	0.04295	947.372**	−327.925
Longitude	0.1929	0.03854	307.122**	−33.325	0.5154	0.00001	919.746**	−328.086
Altitude	0.3035	0.00001	278.665**	−51.576	0.1067	N.S.	938.028**	−327.979
Bio-climates	0.2646	0.00021	303.804**	−33.797	0.2371	0.00554	914.566**	−328.533
Geo-distance	0.2831	0.00215	312.604**	−33.166	0.4896	0.00001	947.372**	−327.925
Bio+Geo			303.803**	−31.797			914.594**	−326.533

**P < 0.01. AIC, Akaike Information Criterion.

### Recent Demographic Change of *Elymus sibiricus*

The significantly negative *Fs* values at the species, QTP, XJ, and NC levels (Table 8) indicated an excess of rare alleles and thus a recent regional expansion, something that was also proven by the non-significant SSD and H_*Rag*_ values.

**TABLE 8:** Analysis of neutrality and mismatch distribution.

Population	Tajima’s *D* (*P*)	Fu’s *Fs* (*P*)	SSD (*P*)	H_*Rag*_ (*P*)
QTP	1.448 (0.938)	−24.812 (0.000)	0.002 (0.371)	0.006 (0.624)
XJ	0.622 (0.765)	−7.528 (0.001)	0.006 (0.075)	0.028 (0.109)
NC	−0.736 (0.264)	−4.972 (0.020)	0.022 (0.197)	0.058 (0.171)
Species	0.893 (0.851)	−24.869 (0.000)	0.003 (0.287)	0.009 (0.120)

*SSD, sum of square deviations; H_Rag_, Harpending’s raggedness index; P, significance.*

### Ecological Niche Modeling of *Elymus sibiricus*

**FIGURE 4:**
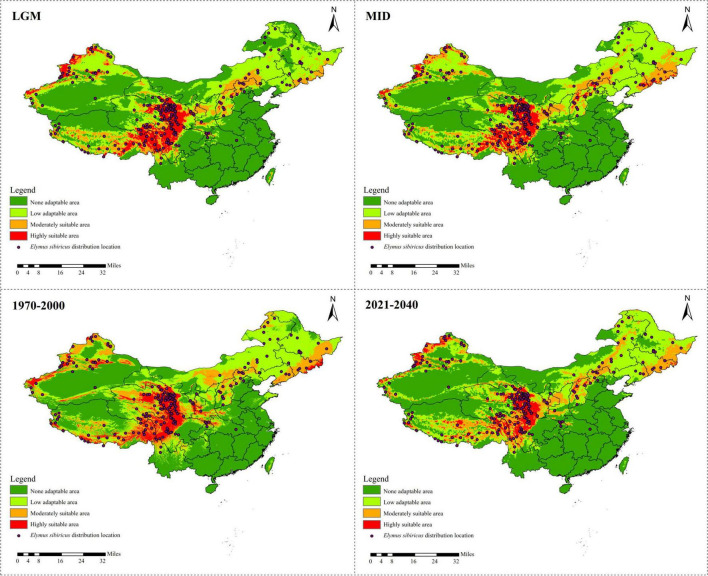
Ecological niche modeling of *Elymus sibiricus* in China showing its distribution dynamics based on MaxEnt model.

A total of 218 referable location records were applied to model the ecological distribution of *E. sibiricus* in China. The results show that the Qinghai–Tibet Plateau is the dominant suitable distribution area of *E. sibiricus*, followed by Xinjiang (Figure 4). There was a clear ecological niche expansion from the LGM to the MID scenario, with subsequent further expansion from MID to the recent past (1970–2000), and an expected distribution area reduction in the near future (2021–2040) (Figure 4 and Supplementary Table 1). As presented in Supplementary Table 2, Bio 1 (annual mean temperature) was estimated to be the most contributing bio-climatic variable to the MaxEnt modeling approach of all the four above-mentioned scenarios, followed by Bio 8 (mean temperature of the wettest quarter) in LGM, 1970–2000 and 2021–2040, and Bio 10 (mean temperature of the warmest quarter) in MID. It is worth noting that Bio 1, Bio 8, and Bio 10 are all temperature-related environmental factors.

### The Population History of *Elymus sibiricus* in China

**FIGURE 5:**
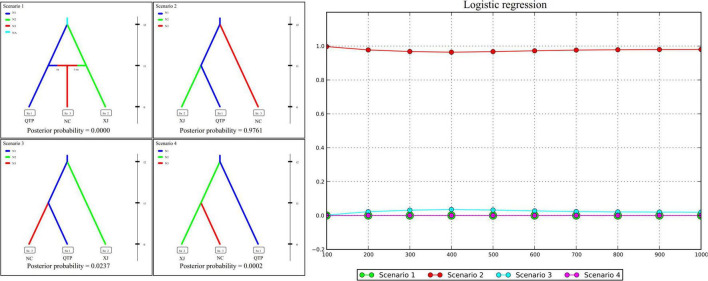
Approximate Bayesian computation (ABC) modeling results of four assumed scenarios and their corresponding posterior probabilities.

Genetic diversity, population structure, gene flow pattern, and ecological niche dynamics suggest the QTP as the diversity center of *E. sibiricus* in China. Four possible scenarios were assumed to examine the population history of *E. sibiricus* based on the ABC modeling approach. The best-fitting scenario (namely, Scenario 2), which assumed QTP as the common ancestor and then the separation of NC and XJ, was adopted based on its maximal posterior probability (0.9761 ± 0.0929, Figure 5). This suggests QTP is the common ancestor population of *E. sibiricus* in China.

## Discussion

### Qinghai-Tibet Plateau Represents the Potential Diversity Center of *Elymus sibiricus* in China

As the largest and highest plateau in the world, the unique geological history and rich natural environmental variation of the QTP have bred numerous unique and rare animal and plant species. The area is thus considered an important alpine biodiversity hotspot and ecological shelter. Most species or genera endemic to the QTP are believed to have originated *in situ* or in adjacent areas (Wang et al., 2007; Zhang et al., 2011; Zhou et al., 2013). Species diversity has been widely explored across the QTP to reveal specific phylogenetic patterns or to find out corresponding diversity hotspots. Yu et al. (2019) characterized the Hengduan Mountains as a genetic diversity center and identified nine evolutionary hotspots across the QTP based on published plastid DNA sequences from 60 plant species occurring in the QTP. In this study, 137 *E. sibiricus* accessions covering the main distribution areas in China were used for the analysis. The QTP group was characterized as having the most abundant haplotype number and diversity based on cpDNA (Table 3) and the highest genetic diversity (Na, Ne, I, and h) based on cpSSR (Table 2). Similar results for *E. sibiricus* were also obtained using nuclear SSR by Xiong et al. (2021), which revealed the highest genetic diversity in the QTP population. In addition, the QTP-based haplotypes had a more ancient divergence time (Figure 2), and there was a obviously higher monodirectional gene flow from the QTP group to the non-QTP group (Figure 3). The ecological niche modeling results also indicate the QTP as the dominant suitable distribution area of *E. sibiricus* (Figure 4). Furthermore, the ABC modeling results reconfirmed the ancestor population status of the QTP (Figure 5). All the above-mentioned results suggest that the QTP is the potential genetic founder and diversity center of *E. sibiricus* in China.

### Divergence Pattern of *Elymus sibiricus* May Be Attributed to Isolation by Distance and Isolation by Environment

Genetic differentiation (F_*st*_) represents the allele frequency differences between the populations. On the one hand, isolation by distance (IBD), which is caused by remote geographical distances or genetic geographic barriers, such as rivers, mountains, and deserts, is known to weaken intrapopulation migration, mating, and gene flow (N_*m*_), thus resulting in a significant phylogenetic structure. On the other hand, isolation by environment (IBE), which relates to habitat heterogeneity, is also believed to be responsible for population divergence. In previous studies, IBD or IBE patterns were found in several *Elymus* species, such as *E. tangutorum* (*r* = 0.312, *P* = 0.001) (Wu et al., 2019), *E. nutans* (*r* = 0.433, *P* = 0.010) (Chen et al., 2013), *E. excelsus* (r = 0.202, *P* < 0.01) (Xiong et al., 2019), and *E. athericus* (r = 0.095, *P* = 0.04) (Bockelmann et al., 2003). Considering the self-pollination and vegetative propagation character of *E. sibiricus*, as well as the matrilineal inheritance of chloroplast DNA, there is expected to be no difference between QTP and its propagated population, namely, XJ and NC. Due to the large geographical distance and huge changes in environmental conditions between populations, however, a certain degree of genetic drift and local adaption is foreseeable. This is confirmed by the high F_*st*_ values (Table 4), obvious phylogeographical structures (Table 6), and significant IBD and IBE patterns (Table 7).

### Ecological Niche and Genetic Diversity Dynamics of *Elymus sibiricus* Point to Its Conservation Applications in China

Genetic diversity is the basis of species survival and evolution (Hughes et al., 2008). Adequate genetic diversity levels help species withstand the negative effects of climate change, inbreeding, habitat fragmentation, and loss, thereby maintaining fitness and providing the ability to evolve, thus contributing to the persistence of the species (Matesanz et al., 2017). The main factors affecting the diversity level include climatic conditions, breeding system, life type, human activity, and others (Sun et al., 2011). Therefore, understanding the genetic diversity patterns of a species and their relationship to ecological characteristics can help us better utilize and conserve genetic resources (Bockelmann et al., 2003). Our results show the highest diversity level of the QTP group (Tables 2, 3) and point to its crucial status as a natural germplasm gene bank. However, efforts are also necessary to protect the populations of XJ and NC.

*Elymus sibiricus*, a cool-season perennial grass species with excellent cold adaptability, is believed to be able to endure −40°C temperatures in winter (De et al., 2011). However, high temperature prevents its further spreading (Figure 4). As a result, the main distribution region of *E. sibiricus* is restricted to certain temperate or cold zones or to high mountains, for example, the QTP, XJ, and NC regions in China (Figure 4; Xin, 2018). The significant negative *Fs* (Table 8) value indicates the rapid population expansion of *E. sibiricus* after a bottleneck, which is also proven by the continuous ecological niche expansion from the LGM (about 22,000 years ago) to the recent past (1970–2000). However, the ecological niche distribution in the near future (2021–2040) is predicted to undergo a dramatic shrinkage (Figure 4 and Supplementary Table 1). This tendency is consistent with the global temperature changes, namely, the temperature decreasing from the LGM to the late nineteenth century, followed by the well-known warming occurring in the past 40 years (Westerhold et al., 2020). To cope with the unprecedented recovery from warming (You et al., 2020), steps must be taken to improve the heat tolerance of *E. sibiricus* through *in situ* conservation and breeding measures, such as molecular marker-assisted selective breeding, crossbreeding, genomic selection breeding, and others.

## Conclusion

The QTP was identified as the diversity center of *E. sibiricus* in China due to its high diversity, obvious monodirectional gene flow (from QTP to non-QTP populations), strongly concentrated ecological niche distribution, and ancient phylogenetic pattern. In spite of the confused genetic structure and haplotype network, significant population divergence was found in this study, which could be attributed to IBD and IBE patterns. The predicted ecological niche shrinkage of *E. sibiricus* in the near future indicates that measures must be undertaken to deal with its intolerance to high temperatures, particularly continued exposure to warmer temperatures.

## Materials and Methods

### Sampling and DNA Extraction

Matured spike samples from a single plant were collected from 23 natural populations, covering the main geographical distribution of *E. sibiricus* in China (Supplementary Table 3 and Supplementary Figure 3). Fresh leaves were sampled from 5 to 6 greenhouse-grown seedlings for each population, with a total of 137 accessions. The sampled leaves were immediately dried with silica gel until DNA extraction was carried out. To avoid collecting the same individual repeatedly due to the clonal reproduction character of *E. sibiricus*, all the samples within a population were spaced at least 50 m apart (Ma et al., 2008; Zhang et al., 2017). The geographical information, including latitude, longitude, and altitude, was collected with a GPS at each population site (Supplementary Table 3). The total genomic DNA of each sample was extracted by applying a modified CTAB procedure (Doyle, 1991). The DNA quality was detected by running the DNA samples on 0.8% agarose gel. Template DNA was diluted to 20 ng/μl and then stored at −20°C until further use.

### cpSSR Procedure

Six polymorphic cpSSR primer pairs (Es-cpSSR1–Es-cpSSR6, Supplementary Table 4) that were developed based on the chloroplast (cp) genome sequences of *E. sibiricus* (NCBI accession numbers: MH732740.1 and NC058919.1) and its related species *Pseudoroegneria libanoticus* (NCBI accession number: KX822019.1) were adopted for population amplification. Polymerase chain reaction (PCR) was carried out in a 15-μl reaction mixture that consisted of 3 μl of template DNA, 7.5 μl of dye-free Taq Mix, 0.4 μl each of forward and reverse primers, 0.2 μl of Taq DNA polymerase, and 3.5 μl of ddH_2_O. The PCR reaction conditions followed those of Ma et al. (2008) with minor modifications. The concentration of PCR products was adjusted to 100 ng/μl, with a minimum sample volume of 24 μl. The adjusted PCR products were then separated on an AATI Fragment Analyzer (Advanced Analytical Technologies, Ames, IA, United States) using DNF-900-K0500 (35–500 bp) as the internal marker. For genotyping, alleles were sized using the PROSize 3.0 software. The genotyping data are presented in Supplementary Table 5.

### cpDNA Procedure

Five cpDNA sequences (*matK*, *rbcL*, *trnY*-*GUA*∼*trnD*-*GUC*, *atpH*∼*atpF*, and *trnT*-*UGU*∼*rps4*, Supplementary Table 4), which were identified as hotspot regions by mVISTA blasting (Supplementary Figure 2) based on the above-mentioned cp genomes, were chosen for amplification and sequencing. The PCR reaction conditions during the entire experiment were set to be consistent with the cpSSR procedure. PCR products were visualized on 1% agarose gels and then purified using a TIANquick Midi Purification Kit (Tiangen Biotech, Beijing, China). DNA sequencing was performed on the ABI 3100 Genetic Analyzer (Applied Biosystems, Waltham, CA, United States) with both upstream and downstream primers. The sequences were edited and aligned manually using the BioEdit software (Hall, 1999). The obtained sequences were uploaded as mentioned in Supplementary Table 6.

### Genetic Diversity Calculation

We artificially divided all the tested accessions into three new groups, namely, Qinghai–Tibet Plateau (QTP), Xinjiang (XJ), and North China (NC), according to the ecological niche prediction results and corresponding geographic features (Supplementary Table 3). All further analyses were performed based on these three groups. For the diversity analysis of cpSSR, GenAlEx 6.5 (Peakall and Smouse, 2006) was used for the calculation of the number of alleles per locus (N_*a*_), the number of effective alleles per locus (N_*e*_), Shannon’s information index (I), and Nei’s genetic diversity (h) of both the populations and primers. At the same time, the PIC index was calculated using PICcalc (Nagy et al., 2012) to reflect the effectiveness of each primer. For cpDNA, DnaSP v5 (Librado and Rozas, 2009) was used for the estimation of the number of segregating sites (S), the number of haplotypes (H), haplotype diversity (Hd), and nucleotide diversity (π). WinArl35 (Excoffier and Lischer, 2010) was used to detect if there was a demographic change by the calculation of Tajima’s *D*, Fu’s *Fs*, sum of square deviations (SSD), and Harpending’s raggedness index (H_*Rag*_). Typically, the significantly negative Tajima’s *D* or Fu’s *Fs*, as well as the non-significant mismatch parameters SSD and H_*Rag*_, indicate potentially historical demographic expansions (Liu L. et al., 2017). We combined three non-coding cpDNA fragments into a single dataset, that is, Es-cpDNA3+4+5. All further analyses of cpDNA were performed based on the three cpDNA regions: *matK*, *rbcL*, and Es-cpDNA3+4+5.

### Population Structure

The STRUCTURE (Evanno et al., 2005) software was applied to determine the genetic membership coefficient of all the individuals for the cpSSR dataset assuming a cluster number from 1 to 4 (K = 1–4). All the other parameters were similar to those of Ma et al. (2008) with minor adjustments. The optimal K value was obtained based on the STRUCTURE HARVESTER (Earl and Vonholdt, 2012) online program *via* ΔK estimation. Principal component analysis (PCA) was conducted in GenAlEx 6.5 (Peakall and Smouse, 2006). Analysis of molecular variance (AMOVA) and calculation of pairwise F_*st*_ value were conducted in WinArl35 (Excoffier and Lischer, 2010) to examine the genetic differentiation between populations for both the cpSSR and the cpDNA datasets. After haplotype distribution was estimated as described earlier, haplotype networks were constructed *via* POPART (Leigh and Bryant, 2013) for the detection of genealogical relationships. The phylogenetic tree of haplotypes was constructed using MEGA 5 (Tamura et al., 2011). To test if there exists a phylogeographic structure, N_*st*_ and G_*st*_ values were calculated using PERMUT (Petit and Grivet, 2002) based on 10,000 permutations. Differences between G_*st*_ and N_*st*_ were estimated using u-statistic, a significantly higher N_*st*_ represents the existence of a phylogeographical structure (Petit and Grivet, 2002). In addition to F_*st*_, gene flow (Nm) is another determinant of population genetic structure (Sexton et al., 2014). Historic pairwise population Nm and effective population size (Θ) were calculated using the software Migrate-n with parameters described by Zeng et al. (2018).

### Isolation by Distance and Isolation by Environment

Genetic drift usually results in more differentiated populations at greater distances assuming no dispersal and selection, namely the so-called IBD. However, the IBD pattern neglects the possibility of environment-driven or hindered gene flow, that is, IBE. Thus, the combination of IBD and IBE could better explain the general patterns of gene flow across environments versus geographic distance (Sexton et al., 2014). In this study, IBD and IBE were detected based on the Mantel test (Diniz-Filho et al., 2013). Specifically, the genetic distance matrix and environment distance matrix were applied for the calculation of the correlation coefficient and *P*-value. The environmental data (19 bio-climate parameters, Supplementary Table 3) were extracted by DIVA-GIS (Hijmans et al., 2004) from a climate dataset downloaded from the WorldClim online website^1^ based on the latitude and longitude of the samples. In addition, the generalized linear mixed modeling (GLMM) approach was also used to reconfirm whether the population’s genetic structure was driven by environmental variables. The analysis was implemented using the R package MCMC_*GLMM*_ (Hadfield, 2010) following the scripts available in Lexer et al. (2014). In total, five models, including Latitude, Longitude, Altitude, Bio-distance (Bio-climate distance), Geo-distance (geographical distance), and “Bio + Geo,” were tested. The Akaike Information Criterion (AIC) was calculated to compare the models and determine the role of IBD versus IBE in the population structure of *E. sibiricus*.

### Ecological Niche Prediction

MaxEnt (Merow et al., 2013) model was applied to predict the ecological niche distribution of *E. sibiricus* in China under the maximum entropy model. Besides our own 137 locations, another 81 records sourced from the Chinese Virtual Herbarium^2^ were also considered, thus totaling to 218 records (Supplementary Table 7). Environmental variables (19 bios) under four scenarios were collected from the WorldClim TIF layer, that is, the Last Glacial Maximum (LGM, about 22,000 years ago), Mid-Holocene (MID, about 6,000 years ago), 1970–2000 (recent past), and 2021–2040 (the near future). To avoid multicollinearity, variance inflation factors (VIFs) were calculated. Bio-variables having a high VIF value (VIFs > 10) were excluded from further analysis. During modeling, a maximum of 10,000 iterations and a bootstrap value of 10 were set. Of the records, 75% were randomly selected for modeling and another 25% were used for validation. Response curves and Jackknife measures were applied to analyze the environmental factors affecting the distribution of *E. sibiricus*. For more detailed setting parameters, we referred to Liu L. et al. (2017). ArcGIS (Scott and Janikas, 2010) was used for the reclassification and visualization of potential distributions. We classified the suitable degree into four levels based on the predicted possibility: 0–5% is a non-adaptable area, 6–50% is a low adaptable area, 51–95% is a moderately suitable area, and 96–100% is a highly suitable area. In addition, the distribution proportion of the above-mentioned four types of suitable areas was calculated based on the corresponding picture element number.

### Demographic History Inferred From Approximate Bayesian Computation Modeling

Four hypothesized scenarios of population history were assumed and tested based on the combined cpDNA and cpSSR datasets. Analysis was performed using approximate Bayesian computation (ABC) with the software DIYABC v. 2.1 (Cornuet et al., 2014). In our assumption, Scenario 1 described the lineage divergence of population QTP (with the effective population size of N1) and XJ (N2) from the common ancestor (NA) at time t2, and the subsequent admixture event between QTP (ra) and XJ (1-ra) which gives birth to population NC (N3) at t1. Scenario 2 assumes that the NC and XJ separated from an ancestor population QTP at t2 and t1 successively. In Scenario 3, XJ and NC separated from ancestor QTP at t2 and t1 successively. In Scenario 4, XJ first separated from ancestor QTP at t2, followed by the separation of NC from XJ at t1 (Figure 5). In reference to Zhang et al. (2019) and Garg et al. (2020), 1% of simulated datasets were chosen for optimal scenario selection by logistic regression. In addition, 1% of the simulated datasets closest to the observed data set were also set to determine the best model and to estimate posterior parameter distributions.

## Data Availability Statement

The datasets presented in this study can be found in online repositories. The names of the repository/repositories and accession number(s) can be found in the article/Supplementary Material.

## Author Contributions

XM, SB, DL, and JY conceived the ideas. XL, YLX, and QY contributed to the sampling procedure. YX, XL, and XS conducted the data analyses. YX and YLX wrote the manuscript. All authors have read and approved the final manuscript.

## Conflict of Interest

The authors declare that the research was conducted in the absence of any commercial or financial relationships that could be construed as a potential conflict of interest.

## Publisher’s Note

All claims expressed in this article are solely those of the authors and do not necessarily represent those of their affiliated organizations, or those of the publisher, the editors and the reviewers. Any product that may be evaluated in this article, or claim that may be made by its manufacturer, is not guaranteed or endorsed by the publisher.

## Footnotes

1
https://www.worldclim.org/

2
http://www.cvh.ac.cn/

## Supplementary Material

Supplementary Figure 1 ΔK estimation based on the structure harvester of cpSSR.

Supplementary Figure 2 Hotspot regions identified by mVISTA blasting based on chloroplast genome sequences of *Elymus sibiricus* and *Elymus nutans*.

Supplementary Figure 3 Geographic distribution map of *Elymus sibiricus* accessions used in this study.

Supplementary Table 1 Proportion statistics of ecological niche distribution of *Elymus sibiricus*.

Supplementary Table 2 Statistics of the contribution rate of 19 bio-climatic variables to the ecological niche distribution of *Elymus sibiricus*.

Supplementary Table 3 Sampling sites, sample size, and bio-climate information of 137 *Elymus sibiricus* accessions.

Supplementary Table 4 Basic information of studied primers.

Supplementary Table 5 cpSSR genotype records.

Supplementary Table 6 cpDNA sequences of *matK*, *rbcL*, and Es-cpDNA3+4+5.

Supplementary Table 7 *Elymus sibiricus* records used for ecological niche modeling.
