# Supplementary material for: Molecular Phylogeography and Intraspecific Divergences in Siberian Wildrye (Elymus sibiricus L.) Wild Populations in China, Inferred From Chloroplast DNA Sequence and cpSSR Markers
Source: Front Plant Sci. 2022 May 19;13:862759. doi: 10.3389/fpls.2022.862759 (PMC9161273; doi:10.3389/fpls.2022.862759)
Supplement: Supplementary Figure 1 — ΔK estimation based on the structure harvester of cpSSR. [file Data_Sheet_1.ZIP › Supplementary Material/Table S1.docx]

Table S1. Proportion statistics of ecological niche distribution of *E. sibiricus*

| **Degree\Scenario** | **LGM** | **MID** | **1970-2000** | **2021-2040** |
| --- | --- | --- | --- | --- |
| Highly adaptable area | 0.0732 | 0.0662 | 0.0822 | 0.0564 |
| Moderately suitable area | 0.1143 | 0.1313 | 0.1560 | 0.1432 |
| Low suitable area | 0.2694 | 0.2890 | 0.2891 | 0.2438 |
| Sum of distribution area | 0.4569 | 0.4865 | 0.5273 | 0.4434 |
| None suitable area | 0.5431 | 0.5135 | 0.4727 | 0.5566 |
