# Supplementary material for: Molecular Phylogeography and Intraspecific Divergences in Siberian Wildrye (Elymus sibiricus L.) Wild Populations in China, Inferred From Chloroplast DNA Sequence and cpSSR Markers
Source: Front Plant Sci. 2022 May 19;13:862759. doi: 10.3389/fpls.2022.862759 (PMC9161273; doi:10.3389/fpls.2022.862759)
Supplement: Supplementary Figure 1 — ΔK estimation based on the structure harvester of cpSSR. [file Data_Sheet_1.ZIP › Supplementary Material/Table S2.docx]

**Table S2.** Statistics of contribution rate of 19 bioclimatic variables to the ecological niche distribution of *E. sibiricus*

| **Variable** | **Code** | **LGM** | **MID** | **1970-2000** | **2021-2040** |
| --- | --- | --- | --- | --- | --- |
| bio1 | Annual Mean Temperature | 28.6 | 30.8 | 23.7 | 23 |
| bio2 | Mean Diurnal Range^a^ | 1.1 | 1.4 | 2 | 3.2 |
| bio3 | Isothermality^b^ | 0.7 | 0.5 | 2.1 | 1.5 |
| bio4 | Temperature Seasonality^c^ | 2.7 | 3.2 | 2.1 | 3.2 |
| bio5 | Max Temperature of Warmest Month | 9.9 | 9 | 9.5 | 10.4 |
| bio6 | Min Temperature of Coldest Month | 3.1 | 2.5 | 1.2 | 1.4 |
| bio7 | Temperature Annual Range^d^ | 1.6 | 1.4 | 2.2 | 1.5 |
| bio8 | Mean Temperature of Wettest Quarter | 16.2 | 9.1 | 13.5 | 14.4 |
| bio9 | Mean Temperature of Driest Quarter | 1.4 | 2 | 5.3 | 2.2 |
| bio10 | Mean Temperature of Warmest Quarter | 8.5 | 15.3 | 12.5 | 12 |
| bio11 | Mean Temperature of Coldest Quarter | 3.3 | 4.8 | 3.8 | 4.3 |
| bio12 | Annual Precipitation | 10.1 | 5.7 | 9.2 | 7.2 |
| bio13 | Precipitation of Wettest Month | 0.9 | 0.8 | 1 | 1.5 |
| bio14 | Precipitation of Driest Month | 0.4 | 0.2 | 0.3 | 0.9 |
| bio15 | Precipitation Seasonality^e^ | 5.6 | 6.9 | 6.6 | 5 |
| bio16 | Precipitation of Wettest Quarter | 0.7 | 1 | 0.5 | 1.3 |
| bio17 | Precipitation of Driest Quarter | 2 | 3 | 1.1 | 1.5 |
| bio18 | Precipitation of Warmest Quarter | 1.4 | 0.8 | 2.5 | 3.7 |
| bio19 | Precipitation of Coldest Quarter | 1.8 | 1.6 | 1 | 1.7 |

^a^, Mean Diurnal Range = Mean of monthly (max temp - min temp). ^b^, Isothermality = (BIO2/BIO7) (×100). ^c^, Temperature Seasonality refers to its standard deviation ×100. ^d^, Temperature Annual Range = BIO5-BIO6. ^e^, Precipitation Seasonality refers to its coefficient of variation.
