# Supplementary material for: Molecular Phylogeography and Intraspecific Divergences in Siberian Wildrye (Elymus sibiricus L.) Wild Populations in China, Inferred From Chloroplast DNA Sequence and cpSSR Markers
Source: Front Plant Sci. 2022 May 19;13:862759. doi: 10.3389/fpls.2022.862759 (PMC9161273; doi:10.3389/fpls.2022.862759)
Supplement: Supplementary Figure 1 — ΔK estimation based on the structure harvester of cpSSR. [file Data_Sheet_1.ZIP › Supplementary Material/Table S3.docx]

**Table S3.** Sampling sites, sample size and bio-climate information of 137 *Elymus sibiricus* accessions

| **Region** | **Population** | **N** | **Latitude** | **Longitude** | **Altitude (m)** | | **Bio1** | | **Bio2** | | **Bio3** | | **Bio4** | **Bio5** | **Bio6** | |
| --- | --- | --- | --- | --- | --- | --- | --- | --- | --- | --- | --- | --- | --- | --- | --- | --- |
| QTP | XZ01 | 6 | 29.7367 | 98.7231 | 3926 | 4.3542 | | 13.4583 | | 45.6215 | | 607.1448 | | 17.5 | -12.0 |  |
|  | XZ02 | 6 | 29.9838 | 96.6705 | 4229 | 2.9125 | | 13.3083 | | 44.3611 | | 615.4382 | | 16.4 | -13.6 |  |
|  | XZ03 | 6 | 29.5407 | 96.7781 | 4388 | 2.0250 | | 12.7500 | | 44.5804 | | 591.9210 | | 14.8 | -13.8 |  |
|  | GS01 | 6 | 35.2065 | 102.6747 | 3100 | 2.2083 | | 14.3167 | | 38.4857 | | 782.5304 | | 18.3 | -18.9 |  |
|  | GS02 | 6 | 34.4964 | 102.6788 | 3266 | 2.0708 | | 14.8917 | | 40.1393 | | 753.0346 | | 17.9 | -19.2 |  |
|  | GS03 | 6 | 34.9415 | 102.9164 | 3061 | 2.5792 | | 14.4583 | | 38.9712 | | 772.5032 | | 18.6 | -18.5 |  |
|  | SC01 | 6 | 31.8800 | 102.6200 | 3974 | 1.5542 | | 14.2917 | | 43.4397 | | 639.4794 | | 15.7 | -17.2 |  |
|  | SC02 | 6 | 33.5830 | 103.2290 | 3246 | 3.0458 | | 14.9250 | | 41.2293 | | 715.9528 | | 18.6 | -17.6 |  |
|  | SC03 | 6 | 32.7623 | 102.0556 | 3504 | 3.5333 | | 16.1500 | | 44.7368 | | 691.3864 | | 18.9 | -17.2 |  |
|  | QH01 | 6 | 36.4411 | 101.9703 | 2493 | 5.2958 | | 13.8083 | | 35.8658 | | 869.3165 | | 22.6 | -15.9 |  |
|  | QH02 | 6 | 33.6333 | 97.1928 | 4418 | -1.7792 | | 14.3917 | | 40.0882 | | 785.1996 | | 14.5 | -21.4 |  |
|  | QH03 | 6 | 37.2814 | 97.3767 | 2907 | 4.2167 | | 14.5333 | | 34.5210 | | 1004.1829 | | 23.7 | -18.4 |  |
| XJ | XJ01 | 6 | 43.4925 | 81.0772 | 2083 | 1.6875 | | 12.0083 | | 27.7971 | | 1143.1180 | | 22.1 | -21.1 |  |
|  | XJ02 | 6 | 47.1772 | 89.7817 | 1216 | 2.0292 | | 13.9917 | | 26.7017 | | 1457.0744 | | 26.5 | -25.9 |  |
|  | XJ03 | 5 | 46.5469 | 90.2853 | 1215 | 2.3958 | | 14.1917 | | 27.1351 | | 1440.9726 | | 26.9 | -25.4 |  |
|  | XJ04 | 6 | 43.6833 | 89.6408 | 1441 | 4.1417 | | 13.4667 | | 27.3713 | | 1328.1426 | | 27.5 | -21.7 |  |
|  | XJ05 | 6 | 43.5814 | 87.2964 | 1534 | 4.8333 | | 11.3333 | | 25.4111 | | 1236.8331 | | 26.4 | -18.2 |  |
| NC | NM01 | 6 | 47.1305 | 119.9200 | 1099 | -3.0917 | | 13.9833 | | 25.7520 | | 1521.3837 | | 22.6 | -31.7 |  |
|  | NM02 | 6 | 43.9952 | 118.5562 | 1157 | 1.6667 | | 13.1000 | | 27.5789 | | 1322.9518 | | 24.5 | -23.0 |  |
|  | NM03 | 6 | 42.5328 | 116.2249 | 1391 | 1.2417 | | 12.6833 | | 26.2052 | | 1356.7589 | | 24.0 | -24.4 |  |
|  | HB01 | 6 | 41.5603 | 115.0464 | 1368 | 3.9500 | | 11.5000 | | 25.4989 | | 1304.9051 | | 25.4 | -19.7 |  |
|  | HB02 | 6 | 41.4847 | 115.6447 | 1510 | 2.5958 | | 11.8917 | | 26.1931 | | 1305.6563 | | 24.0 | -21.4 |  |
|  | HB03 | 6 | 41.3561 | 115.8997 | 1611 | 1.8833 | | 11.9667 | | 26.5336 | | 1286.9632 | | 23.1 | -22.0 |  |

| **Region** | **Bio7** | **Bio8** | **Bio9** | **Bio10** | **Bio11** | **Bio12** | **Bio13** | **Bio14** | **Bio15** | **Bio16** | **Bio17** | **Bio18** | **Bio19** |
| --- | --- | --- | --- | --- | --- | --- | --- | --- | --- | --- | --- | --- | --- |
| QTP | 29.5 | 11.4333 | -2.3833 | 11.4333 | -3.3333 | 511 | 117 | 2 | 101.9172 | 313 | 7 | 313 | 9 |
|  | 30.0 | 10.2000 | -3.8667 | 10.2000 | -4.8000 | 586 | 129 | 3 | 96.9377 | 348 | 14 | 348 | 15 |
|  | 28.6 | 9.0500 | -4.3667 | 9.0500 | -5.3500 | 607 | 134 | 3 | 95.1003 | 357 | 15 | 357 | 16 |
|  | 37.2 | 10.6500 | -8.1333 | 11.2500 | -8.1333 | 561 | 112 | 2 | 91.3461 | 306 | 10 | 302 | 10 |
|  | 37.1 | 10.2333 | -7.9000 | 10.7167 | -7.9000 | 611 | 119 | 2 | 89.4013 | 328 | 12 | 321 | 12 |
|  | 37.1 | 10.9333 | -7.6500 | 11.5000 | -7.6500 | 573 | 113 | 2 | 89.9178 | 308 | 10 | 304 | 10 |
|  | 32.9 | 8.9833 | -6.8000 | 8.9833 | -6.8000 | 882 | 160 | 5 | 86.7680 | 461 | 22 | 461 | 22 |
|  | 36.2 | 10.8833 | -6.4500 | 11.2667 | -6.4500 | 662 | 117 | 3 | 83.1283 | 333 | 16 | 321 | 16 |
|  | 36.1 | 11.4000 | -5.6000 | 11.4000 | -5.6000 | 738 | 137 | 3 | 89.5518 | 385 | 16 | 385 | 16 |
|  | 38.5 | 14.4500 | -6.1833 | 15.3833 | -6.1833 | 417 | 88 | 2 | 95.6955 | 238 | 7 | 234 | 7 |
|  | 35.9 | 7.4000 | -10.9000 | 7.4000 | -11.7500 | 443 | 99 | 2 | 102.7948 | 269 | 9 | 269 | 10 |
|  | 42.1 | 15.9500 | -7.6500 | 15.9500 | -8.8333 | 160 | 36 | 2 | 93.8568 | 94 | 7 | 94 | 9 |
| XJ | 43.2 | 12.8000 | -13.6500 | 14.4833 | -13.6500 | 483 | 88 | 8 | 73.9380 | 244 | 29 | 232 | 29 |
|  | 52.4 | 16.8167 | -14.0500 | 18.4833 | -17.3667 | 153 | 27 | 5 | 48.2470 | 61 | 18 | 61 | 20 |
|  | 52.3 | 18.7333 | -13.3667 | 18.7333 | -16.7333 | 164 | 28 | 6 | 45.0335 | 63 | 21 | 63 | 22 |
|  | 49.2 | 19.4667 | -13.1500 | 19.4667 | -13.1500 | 161 | 28 | 3 | 65.0906 | 75 | 11 | 75 | 11 |
|  | 44.6 | 17.2167 | -11.3000 | 19.0667 | -11.3000 | 228 | 40 | 4 | 69.4621 | 108 | 14 | 107 | 14 |
| NC | 54.3 | 14.5667 | -22.8667 | 14.5667 | -22.8667 | 461 | 125 | 6 | 104.4373 | 297 | 19 | 297 | 19 |
|  | 47.5 | 17.2500 | -15.3000 | 17.2500 | -15.3000 | 413 | 132 | 2 | 125.4297 | 301 | 7 | 301 | 7 |
|  | 48.4 | 16.9167 | -16.6167 | 16.9167 | -16.6167 | 365 | 104 | 2 | 112.0658 | 244 | 9 | 244 | 9 |
|  | 45.1 | 19.1167 | -13.0000 | 19.1167 | -13.0000 | 374 | 98 | 3 | 103.7964 | 234 | 10 | 234 | 10 |
|  | 45.4 | 17.7167 | -14.4000 | 17.7167 | -14.4000 | 419 | 110 | 3 | 102.9322 | 262 | 11 | 262 | 11 |
|  | 45.1 | 16.7833 | -14.9000 | 16.7833 | -14.9000 | 456 | 121 | 3 | 103.3986 | 287 | 12 | 287 | 12 |

N, sample size. Bio1, Annual Mean Temperature. Bio2, Mean Diurnal Range (Mean of monthly (max temp - min temp)). Bio3, Isothermality (bio2/bio7) (×100). Bio4, Temperature Seasonality (standard deviation ×100). Bio5, Max Temperature of Warmest Month. Bio6, Min Temperature of Coldest Month. Bio7, Temperature Annual Range (BIO5-BIO6). Bio8, Mean Temperature of Wettest Quarter. Bio9, Mean Temperature of Driest Quarter. Bio10, Mean Temperature of Warmest Quarter. Bio11, Mean Temperature of Coldest Quarter. Bio12, Annual Precipitation. Bio13, Precipitation of Wettest Month. Bio14, Precipitation of Driest Month. Bio15, Precipitation Seasonality (Coefficient of Variation). Bio16, Precipitation of Wettest Quarter. Bio17, Precipitation of Driest Quarter. Bio18, Precipitation of Warmest Quarter. Bio19, Precipitation of Coldest Quarter. The QTP group include 12 nature populations from Xizang (XZ, 3), Gansu (GS, 3), Sichuan (SC, 3) and Qinghai (QH, 3) respectively. The XJ group include five nature populations from Xinjiang. The NC group include six nature populations from Inner Mongolia (NM, 3) and Hebei (HB) respectively.
