# Supplementary material for: Molecular Phylogeography and Intraspecific Divergences in Siberian Wildrye (Elymus sibiricus L.) Wild Populations in China, Inferred From Chloroplast DNA Sequence and cpSSR Markers
Source: Front Plant Sci. 2022 May 19;13:862759. doi: 10.3389/fpls.2022.862759 (PMC9161273; doi:10.3389/fpls.2022.862759)
Supplement: Supplementary Figure 1 — ΔK estimation based on the structure harvester of cpSSR. [file Data_Sheet_1.ZIP › Supplementary Material/Table S4.docx]

**Table S4.** Basic information of studied primers

| **Code** | **Location** | **Gene length / Product size (bp)** | **Motif** | **Upstream Primer (5’-3’)** | **Downstream primer (5’-3’)** | **Tm (℃)** |
| --- | --- | --- | --- | --- | --- | --- |
| Es-cpDNA1 | *matK* | 1540 |  | TCGAAGGGTATTCAGAAAAACATAA | ATCCAAATACCAAATACGCTCAGT | 60 |
| Es-cpDNA2 | *rbcL* | 1432 |  | GAAACTAAAGCAGGTGTTGGATTT | TAATCGATAGTATCTACCGGCTCG | 59 |
| Es-cpDNA3 | *trnY*-*GUA* ~ *trnD-GUC* | 357 |  | AATCTAGGGCTTCGTGAATATGAA | TGGCATACCCATTCTTAAATAGAAC | 59 |
| Es-cpDNA4 | *atpH* ~ *atpF* | 454 |  | TTTCGATTTCGATTAGATACTTTTTTCT | AGATAGGACGAACAAAGAACAAAGTT | 60 |
| Es-cpDNA5 | *trnT*-*UGU* ~ *rps4* | 299 |  | CATTACAGAATACATCGAAATTAAAACTG | GAACAAGAATCTCTTTTTTTCCGA | 59 |
| Es-cpSSR1 | *psaJ* ~ *rpl33* | 256 | (TTC)_4_ | AGGCCTCAGAAATCAGAGCA | GGCCATGAACCTCCTTTTCT | 54 |
| Es-cpSSR2 | *psbH* ~ *rpoA* | 239 | (AAG)_3_(A)_8_ | TCATTCAACCGTGAAGTACCA | GAGAAGCATCTCCCAATTGAT | 54 |
| Es-cpSSR3 | *trnT*-*UGU* ~ *trnL*-*UAA* | 159 | (A)_12_ | TGAATTGCAATAAGCAAGCG | CCATATCCCCATTTCCCTCT | 55 |
| Es-cpSSR4 | *petG* ~ *trnW*-*CCA* | 218 | (T)_10_ | GGGATTATTCGTGACTGCGT | CAAAACCCGATGTCGTAGGT | 55 |
| Es-cpSSR5 | *rpoC1* | 152 | (AAT)_4_ | AAAGCGTCGAGGTATTTGT | TTTGTCTATAAGCTCCCAGTG | 55 |
| Es-cpSSR6 | *trnW*-*CCA* ~ *trnP*-*UGG* | 140 | (TTCA)_3_ | CGTTCTACCGAACTGAACTAA | CAGTAGCGACGAGATCAATTA | 54 |
