# Supplementary figures and images for: Molecular Phylogeography and Intraspecific Divergences in Siberian Wildrye (Elymus sibiricus L.) Wild Populations in China, Inferred From Chloroplast DNA Sequence and cpSSR Markers
Source: Front Plant Sci. 2022 May 19;13:862759. doi: 10.3389/fpls.2022.862759 (PMC9161273; doi:10.3389/fpls.2022.862759)

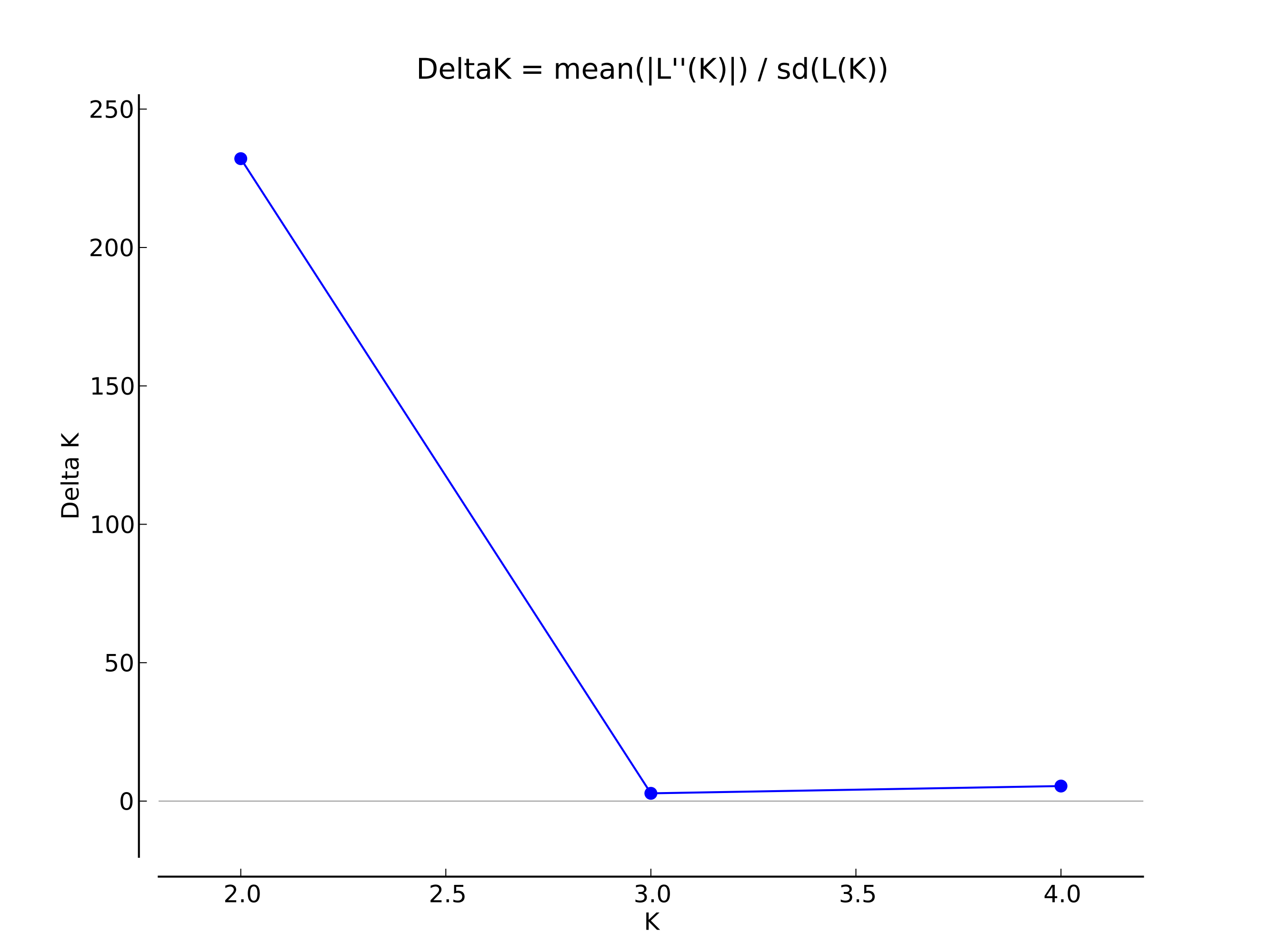

Supplement: Supplementary Figure 1 — ΔK estimation based on the structure harvester of cpSSR. [file Data_Sheet_1.ZIP › Supplementary Material/Fig S1.tif]

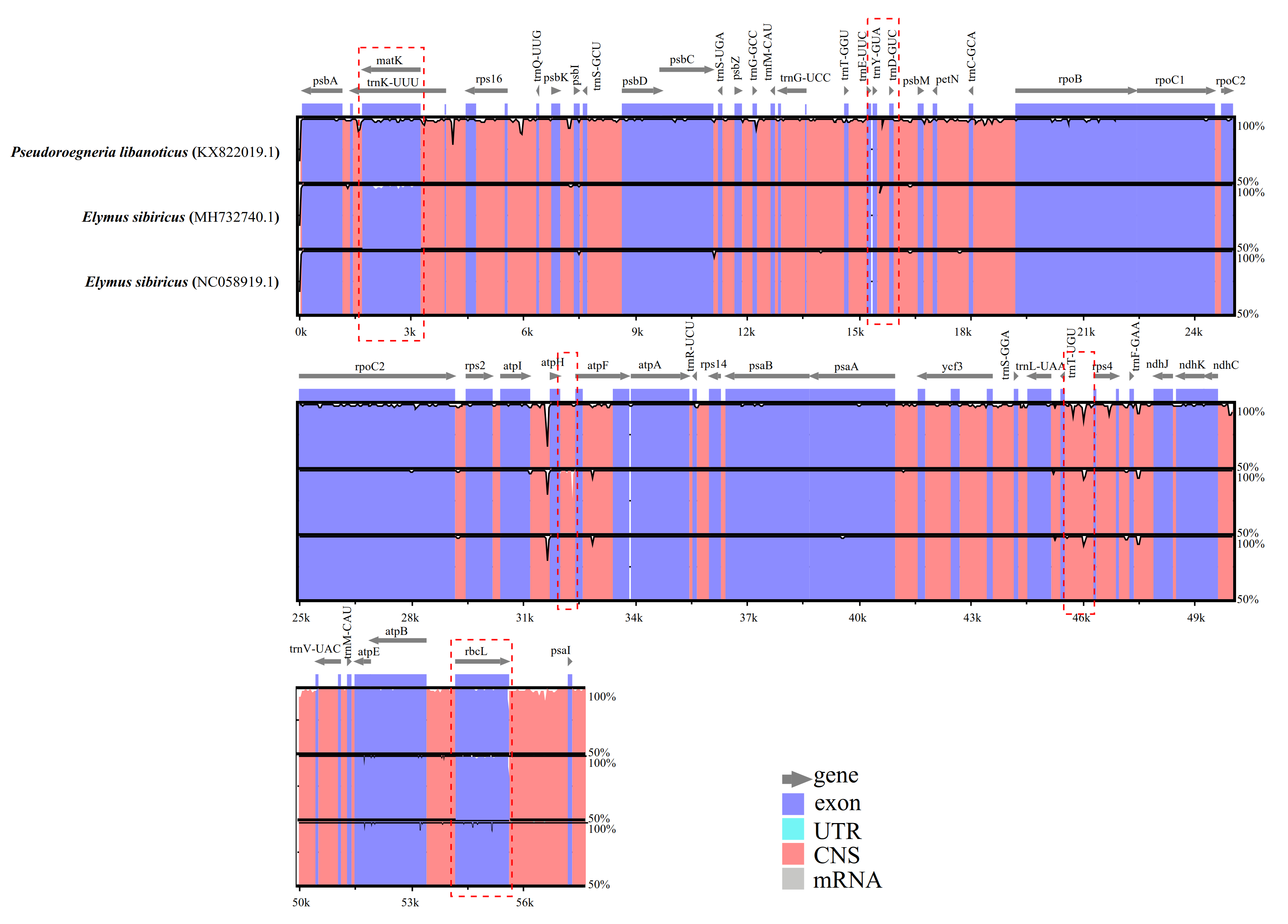

Supplement: Supplementary Figure 1 — ΔK estimation based on the structure harvester of cpSSR. [file Data_Sheet_1.ZIP › Supplementary Material/Fig S2.tif]

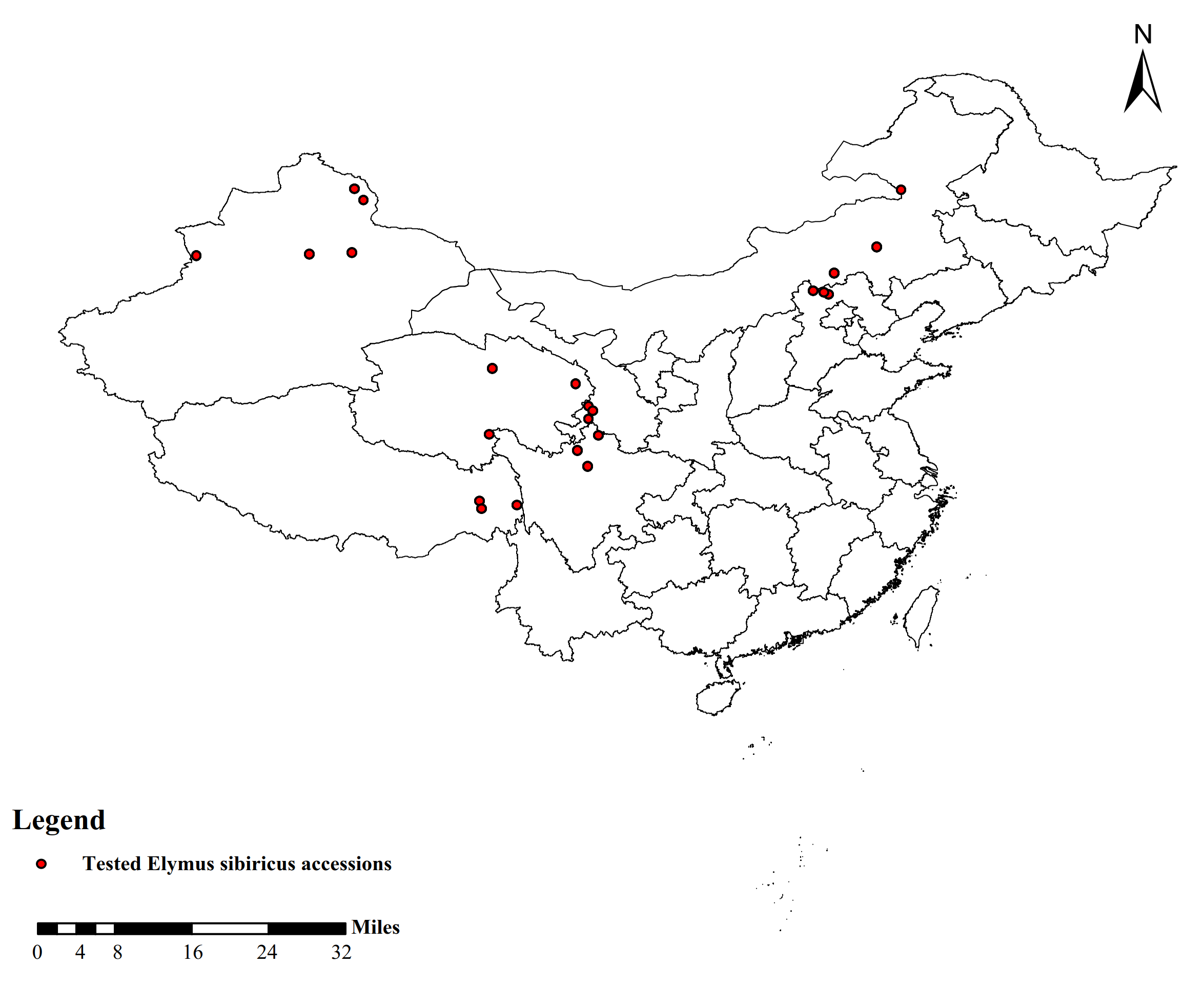

Supplement: Supplementary Figure 1 — ΔK estimation based on the structure harvester of cpSSR. [file Data_Sheet_1.ZIP › Supplementary Material/Fig S3.tif]
